# Systematic evidence maps as a novel tool to support evidence-based decision-making in chemicals policy and risk management

**DOI:** 10.1016/j.envint.2019.05.065

**Published:** 2019-06-26

**Authors:** Taylor A.M. Wolffe, Paul Whaley, Crispin Halsall, Andrew A. Rooney, Vickie R. Walker

**Affiliations:** a Lancaster Environment Centre, Lancaster University, Lancaster, UK; b Yordas Group, Lancaster Environment Centre, Lancaster University, Lancaster, UK; c Division of the National Toxicology Program, National Institute of Environmental Health Sciences, National Institutes of Health, Research Triangle Park, NC, USA; d Evidence-Based Toxicology Collaboration, Johns Hopkins Bloomberg School of Public Health, Baltimore, MD 21205, USA

**Keywords:** Systematic review, Evidence mapping

## Abstract

**Background:**

While systematic review (SR) methods are gaining traction as a method for providing a reliable summary of existing evidence for health risks posed by exposure to chemical substances, it is becoming clear that their value is restricted to a specific range of risk management scenarios - in particular, those which can be addressed with tightly focused questions and can accommodate the time and resource requirements of a systematic evidence synthesis.

**Methods:**

The concept of a systematic evidence map (SEM) is defined and contrasted to the function and limitations of systematic review (SR) in the context of risk management decision-making. The potential for SEMs to facilitate evidence-based decision-making are explored using a hypothetical example in risk management priority-setting. The potential role of SEMs in reference to broader risk management workflows is characterised.

**Results:**

SEMs are databases of systematically gathered research which characterise broad features of the evidence base. Although not intended to substitute for the evidence synthesis element of systematic reviews, SEMs provide a comprehensive, queryable summary of a large body of policy relevant research. They provide an evidence-based approach to characterising the extent of available evidence and support forward looking predictions or trendspotting in the chemical risk sciences. In particular, SEMs facilitate the identification of related bodies of decision critical chemical risk information which could be further analysed using SR methods, and highlight gaps in the evidence which could be addressed with additional primary studies to reduce uncertainties in decision-making.

**Conclusions:**

SEMs have strong and growing potential as a high value tool in resource efficient use of existing research in chemical risk management. They can be used as a critical precursor to efficient deployment of high quality SR methods for characterising chemical health risks. Furthermore, SEMs have potential, at a large scale, to support the sort of evidence summarisation and surveillance methods which would greatly increase the resource efficiency, transparency and effectiveness of regulatory initiatives such as EU REACH and US TSCA.

## 1. Introduction

Systematic review is the epitome of the evidence-based approaches that have revolutionized clinical decision-making. The methodology was developed in response to medical practitioners’ need to distill clear and reliable conclusions about the efficacy of clinical interventions from an evidence base seemingly full of contradiction, heterogeneity and bias (Chalmers et al., 2002; Garg et al., 2008; Higgins and Green, 2011). This need parallels that of chemicals policy; where conclusions regarding the safety of exposure to a chemical substance must be synthesised from a significantly more disparate evidence base (Whaley et al., 2016).

Consequently, interest in the application of systematic review to regulatory decision-making contexts within chemicals policy and wider environmental health is growing. This is evidenced by the increasing number of systematic reviews published in the field (Whaley and Halsall, 2016), the establishment of collaborations and workgroups dedicated to development and dissemination of environmental health systematic review methodology (Morgan et al., 2016; NTP, 2015; Woodruff and Sutton, 2014), and the adoption and use of systematic review by regulatory bodies such as the United States Environmental Protection Agency (US EPA) (EPA, 2018; The National Academies of Sciences, 2017) and World Health Organization (Mandrioli et al., 2018).

Growing interest in systematic review approaches is indicative of the evolutionary journey chemicals regulation follows as it attempts to reconcile past oversights with present day knowledge and mounting future challenges. A number of legacy chemicals released to market under past regulatory workflows persist on the market without risk assessment. Meanwhile, an overwhelming number of new chemicals are presented for assessment each year while awaiting release to market under modern regulatory workflows (European Comission, 2007; Pool and Rusch, 2014). This amounts to increasing strain on regulatory processes, which must operate without a proportionate increase in resource availability. While providing and/or gathering relevant data for new chemicals now forms a vital part of risk assessment, advances in analytical techniques and scientific understanding continue to broaden the scope of this data beyond the realms of traditional *in vivo* toxicity testing. Although vital for compiling a more complete understanding of a chemical’s toxicity, the broad scope and increasing availability of such data presents challenges for decision-makers tasked with handling, appraising and interpreting this data for risk assessment. Failure to have a transparent structure for considering all relevant data appropriate to risk assessment (e.g. a stepwise approach for addressing *in vitro* data following evidence from *in vivo* studies or comprehensive assessment of all *in vitro* data) reduces stakeholder confidence and has the potential to bias regulatory decisions. Studies reporting results amenable to the observer bias of independent assessors, or to the vested interests of non-independent assessors, may be cherry picked from the wider evidence base. Even where all relevant studies are considered, the role that scientific judgement plays in the process of appraisal and interpretation of data can lead to conflicting conclusions between different regulatory bodies (Whaley et al., 2016). Transparency in identifying both the evidence and scientific judgement are critical to establishing trust in decision-making.

Systematic review offers a framework for piecing together this varied data in a transparent and resource efficient manner, such that a more complete picture of toxicity can inform regulatory decision-making. It details methodology for ensuring *all* such data is identified, gathered and considered – preventing cherry picking of studies that only provide part of the complete toxicity profile for a chemical, or that present biased or unrepresentative results. As well as reducing bias, all steps of the methodology are designed to maximise transparency. A well conducted and reported systematic review effectively outlines the research question, the approach taken to address the question, the evidence considered, and the scientific judgement applied to reaching conclusions. Thus, differences across reviews or regulatory bodies can be effectively identified and explained. Considering the results of all relevant studies makes maximum use of existing data and increases the precision of a systematic review’s conclusions. This allows reliable decisions to be made without the commissioning of redundant and repetitive primary research, or conversely identifies specific knowledge gaps at which smart testing strategies can be focused.

Although the aim of systematic review (i.e. to transparently and robustly synthesise all available data in answer to a research question) aligns well with the needs of chemicals policy, conflicts between the practicalities associated with the methodology and those associated with regulatory frameworks hinder their wider uptake, and/or the production of reviews that are of sufficient quality to produce trustworthy results (Kelly et al., 2016; Marshall et al., 2018; Reynen et al., 2018). Key areas of conflict include the time and resource intensity of the systematic review process, the scope of the research questions addressed by the methodology, and the ease with which the output of a systematic review can be accessed, interpreted and updated. Further, the fluid and rapidly expanding nature of scientific research and the chemicals industry creates a constant and pressing need for evidence surveillance, such that regulators can keep apace of the growing body of scientific literature and update regulation accordingly. This challenge demands a responsive and living solution beyond the reach of current systematic review practice.

In this manuscript, we briefly outline systematic review methodology to illustrate its strengths and highlight the transferable barriers which have been suggested as preventing its wider uptake in other fields (Oliver and Dickson, 2016). We discuss how these difficulties may be addressed through the novel implementation of systematic evidence mapping in environmental health. Systematic evidence maps (SEMs) provide a broad and comprehensive overview of an evidence base (Haddaway, Bernes, Jonsson, & Hedlund, 2016; James et al., 2016). They facilitate the identification of trends which can be used to inform more efficient systematic review, or more targeted primary research. The methodology behind SEMs, and how this might be adapted to suit the demands and limitations of regulatory decision-making in chemicals policy is discussed, along with the advantages and future potential of SEMs as a fundamental tool for evidence-informed risk management and decision-making.

## 2. The application of systematic review methods in chemical risk management

The utility and advantages of systematic review methods for advancing chemical risk assessment have been extensively documented elsewhere (Aiassa et al., 2015; Hoffmann et al., 2017; Hooijmans et al., 2012; Rooney et al., 2014; Vandenberg et al., 2016; Whaley et al., 2016; Woodruff and Sutton, 2014). Systematic review provides a transparent and reproducible approach to summarising and critically assessing existing evidence on potential health risks associated with exposure to a chemical substance. These transparent methods serve to document the basis of scientific judgments, minimising the potential for bias and error presented by more traditional narrative approaches in which opinion is not clearly distinguished from evidence.

The key features of a systematic review (Table 1) are:

- a clearly specified research objective - usually captured in a Population-Exposure-Comparator-Outcome (PECO) statement
- a comprehensive search strategy
- screening of the search results - for evidence relevant to addressing the research objective
- extraction of data from included studies - using a prespecified data extraction framework
- critical appraisal of included studies - according to a prespecified set of quality criteria, usually targeting risk of bias
- synthesis of findings from the included studies - using suitable quantitative statistical methods and otherwise qualitative methods as appropriate
- characterisation of confidence in the evidence for the results of the synthesis - according to a prespecified set of criteria
- statement of conclusions - including an assessment of limitations in design and conduct of the review itself.

Specific methodological decisions concerning each of these key features, from definition of the PECO statement to the chosen synthesis approach, are specified in a pre-published protocol.

However, with the methodology’s pursuit of rigor and comprehensiveness comes a significant demand for time and resources. Evidence from medical systematic reviews indicates it takes on average approximately 70 weeks to progress a systematic review from protocol registration in the PROSPERO registry (National Institute for Health Research, 2018) to publication of the final systematic review (Borah et al., 2017). Variance around this average is wide (from 6 to 186 weeks), but the significance of person-hours and planning time prior to protocol registration is not considered in these estimates. More recent analysis of environmental science systematic reviews estimates an average of 164 (full time equivalent) person-days required for completion of systematic reviews (Haddaway and Westgate, 2018). However, in the absence of comparable evidence in the field of chemical risk assessment, these figures agree with anecdotal reports of the average systematic review taking around 12 to 18 months to progress from inception to publication. A significant factor which contributes to the length of the systematic review process is the manual way in which each step of the methodology is conducted. All studies returned by a systematic search strategy are generally screened by human reviewers, in duplicate, one-by-one, before included studies undergo a similarly manual data extraction and critical appraisal step.

Systematic review management software has been developed (e.g. “HAWC: Health Assessment Workplace Collaborative,”, 2013; Covidence, 2019; Evidence Partners, 2019; Science for Nature and People Partnership Evidence-Based Conservation working group, Conservation International, Datakind, 2018; Sciome, 2018; Thomas et al., 2010; CAMARADES-NC3Rs, 2019) to assist human reviewers with maintaining transparency in SRs and with organising the review process. Acknowledging the impedance caused by a review’s manual workload, review management software is beginning to incorporate machine learning as a means of automating labour-intensive tasks (e.g. Evidence Partners, 2019; Science for Nature and People Partnership Evidence-Based Conservation working group, Conservation International, Datakind, 2018; Sciome, 2018; CAMARADES-NC3Rs, 2019). Automation has the potential to result in significantly reduced workloads and subsequent demands for time and resources (Mara-eves et al., 2015). Pending further advances, the time and resource demands of systematic review are at conflict with the intense time/resource pressure under which regulatory processes must operate (Innvaer et al., 2002; Oliver and Dickson, 2016).

Also at conflict with the demands of regulatory decision-making is the narrow scope of systematic reviews, which are designed to address a specific and clearly defined objective or research question. To ensure a manageable, relevant and focused review, suitable research questions are typically closed framed, such that the review can synthesise a single, coherent answer. These closed-framed questions are well suited to the decision-making contexts of medicine (the field from which systematic reviews originate), but may be difficult to apply to chemical risk assessment. The web of interlinked endpoints, potential variation in sensitive populations, uncharacterised low dose effects, and unknown behaviour of a chemical in the environment or in contact with other chemicals can mean that the decision-critical information which can be supplied by a tightly focused research question is often not readily apparent in chemical risk assessment contexts. Even where such a question can be devised, and the answer reached through systematic review, the specificity of the research problem and its resolution are likely to comprise only part of the much broader range of unaddressed decisions and information requirements faced by risk managers.

## 3. Systematic evidence maps for chemical risk management

In light of the time and resource intensity of current systematic review practice, identifying the most informative research questions is important for maximising the value and efficiency of systematic reviews in regulatory decision-making. Investing resources in systematic review as a means of addressing specific research questions is inefficient if there is a lack of data available for answering those questions. Devising specific research questions therefore becomes a reactive process, rather than a proactive one. This is at odds with the goals of chemicals policy, which aims to predict and prevent harm as a result of exposure to chemical substances.

Decision-makers therefore need to monitor and understand the evidence base as a whole – such that emerging trends or issues of potential concern can be identified and investigated in a timely manner. Identifying trends in the evidence base, including evidence clusters and evidence gaps, facilitates the formulation of proactive research questions by relevant stakeholders. Reviewers need not rely on environmental health outcomes becoming infamous or epidemic as an indicator of sufficient evidence for an efficient and valuable synthesis. Instead, trends in the availability of evidence ensure prevention of synthesis attempts for which there is insufficient data (or for which syntheses already exist) and promote the targeting of primary research efforts at evidence gaps. This kind of evidence surveillance has traditionally been the domain of scoping reviews. These reviews are often narrowly focused precursors to systematic reviews. Thus a specific systematic review question has already begun to be framed, and the literature scoped for sufficient data to address/focus it – rather than vice versa (e.g. Bolden et al., 2017). Scoping reviews also typically present their findings in tabular format. This compromises the accessibility of the evidence they scope, and makes them ill-suited for applications beyond determining whether there is sufficient literature to merit a systematic review (Grant and Booth, 2009).

Instead, the introduction of systematic evidence mapping, a methodology recently adapted from the social sciences (Clapton et al., 2009) for environmental management (James et al., 2016), has the potential to facilitate evidence surveillance in a transparent and reproducible manner, providing a broader understanding of the extant evidence base through interactive outputs.

The methodological steps involved in constructing a systematic evidence map are similar to those involved in the initial stages of producing a systematic review (see Table 2, adapted from James et al., 2016) whereby a systematic search strategy is employed to collate evidence, which is subsequently screened for relevance before undergoing data extraction. The key difference between the methodologies comes in the form of their aims and subsequent outputs. Systematic reviews collate a relatively narrow subset of the evidence base to answer a specific research question. Conversely, SEMs do not attempt to answer a specific, closed-framed research question, and are instead guided by much broader research objectives. SEMs collate a sufficiently broad subset of evidence such that many different specific research questions might be formulated from, and addressed with, a single systematic evidence map. SEMs are concerned with characterising the evidence base within a given research area, such that the availability, type and features of the evidence can be clearly mapped and explored through data visualization.

To facilitate this exploration, the output of a SEM takes the form of a queryable database (Clapton et al., 2009; James et al., 2016) as opposed to the lengthy and technical documents which form the main output of a systematic review. The database format allows users to query the evidence base according to their research interests, providing functionality which is void from systematic review documents and their associated static data tables. This format addresses the inability of systematic evidence mappers to predict what the specific research interests of users might be by providing the option to search for, and select, the specific subsets of data relevant to a particular use case.

Whereas systematic reviews present users with select information from included studies (i.e. data relevant to addressing the research question), SEMs aim to extract a broader range of data from included studies and aim to maintain the native format of these data. In this sense, the search and screening process are the steps of SEM methodology most affected by its research objective or context, as the focus of data extraction remains broad regardless. This is in contrast to systematic review, where all steps are heavily influenced by its research question. The data extracted for inclusion in a SEM database can then be flexibly categorised, or “coded” to facilitate comparison of an otherwise heterogeneous evidence base.

Resolution of coding can be adapted to suit the needs of regulators. For example, coding the species under investigation in a study might use categories such as “Sprague-Dawley”, “Rat”, “Rodent” or “Mammal”; or may use all of these categories such that the data can be interrogated in successively deeper levels of detail. As well as facilitating variably resolved interrogation of the evidence base, coding plays a significant role in systematic mapping’s amenability to updating. Use of universal, standardised ontologies for coding, such as the Unified Medical Language System (UMLS) (U.S. National Library of Medicine, 2016), offers a degree of consistency that future users can readily exploit when updating a map (Baker et al., 2018). These ontologies also offer interoperability between SEMs, creating the potential to expand and merge evidence maps – a feature likely to become increasingly attractive as the scope of evidence relevant to assessing toxicity grows along with our understanding of its interconnectedness.

In current practice it is common to present users with SEMs that house only coded information for simplicity and ease of access (e.g. Papathanasopoulou et al., 2016). However, this conflates data extraction with coding. Maintaining the native format of extracted data and applying coding on top of this therefore ensures maximum transparency in SEMs. This additionally promotes the ease with which a map can be updated as advancing scientific understanding calls for coding categories to be redefined. As with systematic reviews, the data extraction and coding steps of a SEM represent a manual workload. Presenting *only* coded data may offer a saving in the resource intensity of the process. However, in maintaining a transparent link between raw extracted data and the code used to categorise it, SEMs offer a gateway to automation – whereby controlled vocabulary ontologies can be used to train machine learning algorithms to automatically identify, extract and code data from the literature.

Pending such advances, the time required to conduct a fit for purpose systematic map in environmental health is uncharacterised. Evidence from the wider environmental sciences (Haddaway and Westgate, 2018) suggests that (on average) systematic maps take longer to complete than systematic reviews. This is due to the generally larger number of studies they manually collate, screen and extract data from. While maps might present a larger upfront cost in terms of time, their multipurpose nature has the potential to offer more long-term resource savings compared to exclusively conducting systematic reviews. This is because a single systematic evidence map may continue to be useful to several different aspects of the regulatory workflow (see Sections 4 and 5 below).

As the purpose of a SEM is to characterise the evidence base, there is no risk of allocating resources to the production of an inconclusive output, as is the case for “empty” systematic reviews (systematic reviews which ask research questions for which there is too little included evidence for them to reach a conclusion or be supportive of a decision). In fact, systematic evidence maps may reduce the resource strain associated with systematic reviews. A SEM’s broad overview of the evidence base allows fast identification of topics for which there is sufficient data to warrant a full systematic review. The SEM itself, if conducted to sufficiently rigorous standards, can even replace the literature search and screening process of a systematic review. As SEMs present all available relevant evidence on a broader topic such as the “health effects of bisphenol-A” (obtained through a systematic but less specific search strategy), filtering this information according to the PECO statement of a systematic review may act in an equivalent manner to approaching the literature with a more focused search strategy in the first instance. The pre-screened nature of this subset is likely to reduce the number of false positive results, facilitating faster syntheses.

As advances in machine learning facilitate more highly resolved data extraction processes, future SEMs may even store enough detail for them to form the basis of meta-analytical syntheses. If all data contained within study reports is extracted and indexed within a SEM, there would be no data required specifically for syntheses which could not be found in the SEM. This would allow SEMs to form the dataset on which meta-analytical and predictive toxicological models are based, the results of which may additionally be incorporated into the SEM itself – facilitating more transparent, resource-efficient and easily updated syntheses.

## 4. Exploring the evidence base with SEMs

Systematic evidence mapping facilitates identification of trends which are informative for many risk management scenarios. To illustrate the flexibility and potential utility of SEMs’ trendspotting capacity, this section highlights the type of data visualization and exploration possible through querying subsets of information in a SEM database. Specifically, “priority setting” (National Academy of Sciences, 1983; Pool and Rusch, 2014), the process by which regulators identify the most pressing chemical substances for assessment and regulation (e.g. from a pool of unassessed legacy chemicals) is presented as context for the exploration of a hypothetical SEM.

Several factors are relevant to prioritizing individual chemicals for assessment, broadly ranging from recorded levels of exposure to evidence for toxicity. Underlying these broad considerations are several more specific factors such as the bio-accessibility of the chemical, the relevance of its toxicity evidence for predicting health risks in human populations etc. In order to make the most efficient use of resources and the systematic review process, decision-makers require access to a means of comparing these features to justify prioritization of a particular chemical for review/risk assessment.

**Fig. 1.**
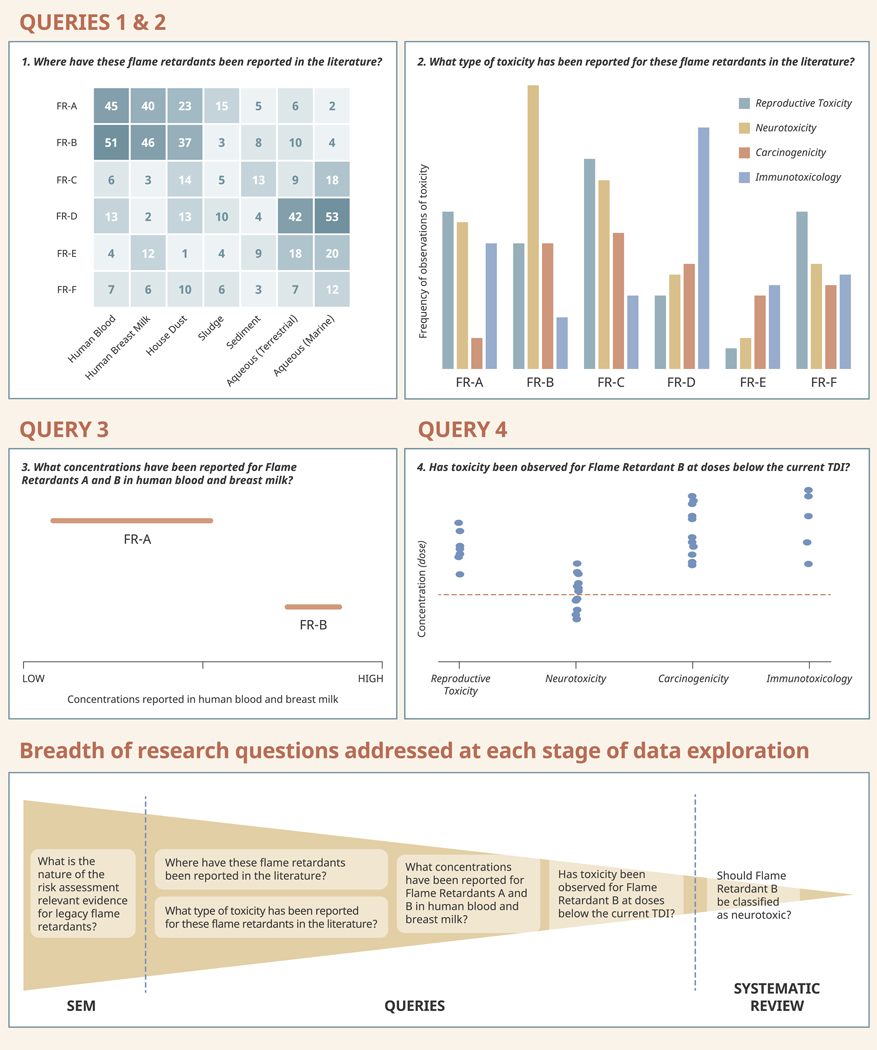
The process of identifying trends and exploring the evidence landscape involves querying the SEM database and visualizing the results of the query. Queries may start by asking broader questions which consider a wider range and volume of data (e.g. Queries 1 and 2). Users may then further explore any trends of interest discovered in the results of these broad queries by running narrower queries which consider a more specific subset of data (e.g. Queries 3 and 4). Data displayed in this Figure have been artificially generated to illustrate a hypothetical use case for SEMs. FR = flame retardant, TDI = tolerable daily intake, SEM = systematic evidence map.

This is the role of a SEM, which may be constructed with the aim of identifying and characterising the risk assessment relevant evidence for a broader group of legacy chemicals, e.g. flame retardants. Once data has been extracted and coded from the literature, the SEM can be explored with a succession of queries of increasingly narrow focus, each considering a narrower subset of the evidence base than the last, such that a research question appropriate for more detailed synthesis is resolved at the end of a process which begins with a very broad research objective. This is illustrated in Fig. 1 using the hypothetical context of priority setting with a group of arbitrary chemicals, in this case flame retardants (FRs) A–F.

Queries 1 and 2 depicted in Fig. 1 explore the frequency with which the literature observes a flame retardant in a coded location category (e.g. human blood, human breast milk, house dust, etc.) and the frequency with which the literature observes an association between a flame retardant and a coded toxicity category (e.g. reproductive toxicity, neurotoxicity etc.). The heatmap visualizing the results of Query 1 shows a comparatively large number of observations of FRs A and B in location categories directly relevant to human populations (i.e. human blood and breast milk). Query 2 clarifies whether these observations require further attention by indicating what kind of toxicity information is available for each flame retardant. The bar chart visualization indicates comparable numbers of observations for most of the flame retardants and types of toxicity but a comparatively large number of observations that associate FR B with neurotoxicity.

Based on (hypothetical) existing evidence, Queries 1 and 2 indicate flame retardants A and B as potential candidates for full assessment. Resolving which to prioritize involves accessing more study-specific information through a series of queries which consider a successively narrow subset of the evidence base. Despite availability of toxicity data, observing flame retardants in human relevant locations might not be concerning if the concentrations observed are negligible. Thus Query 3 examines the range of concentrations reported in the literature for FRs A and B in human blood and breast milk. Visualization of Query 3 indicates a wider range of lower concentrations reported for FR A, compared to a narrower range of higher concentrations for FR B. Query 4 then examines the relevance of these concentrations against the current estimated tolerable daily intake (TDI) for FR B, indicating several observations of toxicity below the current TDI and supporting prioritization of FR B for assessment. Further, the relatively large volume of observations of neurotoxicity may indicate sufficient data available to conduct a systematic review on FR B’s relationship with neurotoxicity.

However, it is important to distinguish the results of SEM queries from synthesis. SEMs only present what has been studied in the literature – they cannot present what has not been studied, and do not always assess the risk of bias of the findings they report. Thus, while a high number of observations of flame retardants A and B in human relevant locations is a valid trend to explore further, it does not necessarily mean that there are fewer of the other flame retardants present in human relevant locations, but rather that there may simply be fewer of these flame retardants studied at all. Identification of such evidence gaps is equally valid for focusing primary research. For example, the relatively high number of observations of reproductive toxicity for FR F, but comparatively low number of observations of this flame retardant in any exposure locations might warrant re-analysis of samples or new exposure studies to verify whether exposure to this substance is of concern.

The SEM is also sufficiently flexible that different trends can be investigated, and different research questions formulated, based on the priorities of regulators. For example, the number of observations in the literature which found FR D in aquatic environments might spur further investigation into the ecotoxicity of this compound. A single SEM exercise therefore makes efficient use of resources in its potential to meet the varied needs of several end users.

## 5. The role of SEMs in wider risk management workflows

In addition to priority setting, SEMs have the potential to fill several roles within wider workflows.

### 5.1. Data gathering

Although evidence synthesis methodology can be considered costly in terms of time and resources, this cost can be dwarfed by the equivalent resource demands associated with conducting primary research relevant to assessing the hazards associated with exposure to a chemical, as illustrated with more established examples in the field of medicine (Glasziou et al., 2006). In an effort to manage these demands, reduce the production of research waste, and comply with principles such as the three Rs (European Chemicals Agency, 2018a, 2018b; National Centre for the Replacement Refinement and Reduction of Animals in Research, 2018), a key first step in many regulatory workflows is the identification and gathering of all pre-existing evidence relevant to a specific risk management decision. This can be illustrated in regulatory frameworks such as the European Union’s REACH (Registration, Evaluation, Authorisation and Restriction of Chemicals) initiative, which requires registrants to make an attempt to identify all available, pre-existing evidence on the hazards associated with the chemical substance under registration (European Chemicals Agency, 2018a, 2018b). Similarly, REACH imposes a “one substance, one registration” policy, whereby all parties with an interest in registration of a substance must share data, minimising repeat testing. Although promoted in guidance documents (European Chemicals Agency, 2016), a lack of a sufficiently robust methodology for finding, collating, housing and reporting these data leads to poor transparency, and therefore does not remove the potential for cherry picking of key studies which may not be representative of the evidence base as a whole.

SEMs have the potential to provide this much needed transparency. The nature of a SEM’s output being a collection of relevant search results, and specific information coded from those results, introduces a greater level of accountability for registrants. Studies are identified by registrants as “key”, “supporting” etc. based on the perceived relevance, adequacy and reliability of the evidence they provide for a specific endpoint, assessed using “sound scientific judgement” (European Chemicals Agency, 2011). These assignments are aided by application of the Klimisch criteria (Klimisch et al., 1997) – a rating methodology criticised for its lack of transparency and failure to consider non-industry sources of evidence (Ingre-Khans et al., 2019). This poor transparency hinders the appraisal of registrants’ choices (e.g. of key study), and the degree to which those choices can be considered representative of the wider evidence base. Using SEM methodology alleviates this issue by requiring registrants to clearly document the efforts of their search and screening process, constructing a database of the pool of evidence considered in their evaluations. Additionally, applying code to the specific extracted study features which influence a decision to assign a study as “key”, “supporting”, “weight-of-evidence” etc. serves to document the basis for these decisions in a structured and queryable way. As registrants submit SEMs at the level of single substances, these efforts can be merged to build a SEM that spans all registered substances. This facilitates appraisal of registrants’ choices of key study in the context of the wider evidence base. The ability to explore trends in the features influencing assignment of key studies may even assist in refining and improving the registration process – as emerging issues or shortcomings can be quickly evidenced.

### 5.2. Problem formulation

Beyond offering improvements in transparency during the data gathering phase, SEMs may be of particular value to the problem formulation stage of regulatory decision-making. Problem formulation is a prerequisite to conducting a chemical risk assessment, identifying an issue of regulatory relevance around which the assessment will be focused (Solomon et al., 2016). These issues can be subtle and difficult to identify at a sufficiently early stage in the field of environmental health, putting the problem formulation process at risk of focusing on issues of lower severity or significance. In implementing a SEM with a broad (lower resolution) coding process, but with a key focus on the hierarchy of coded data and the nature in which this data is related, trends in the evidence base can be effectively and efficiently identified. This allows risk assessors to use these broad, coded parameters to reliably identify problems in need of further assessment, either through secondary syntheses (if the SEM presents a sufficiently large evidence cluster) or primary research (if the SEM indicates an evidence gap).

### 5.3. Read-across

Identifying trends in the evidence base may also play a significant role in read-across applications. Read-across allows the toxicologically relevant properties of a chemical to be inferred by comparison with a structurally similar chemical of known toxicological behaviour (European Chemicals Agency, 2017a). Read-across aligns well with the need to make best use of existing evidence (van Leeuwen et al., 2009), and the storage of data in a related manner within a SEM could allow the identification of appropriate read-across scenarios. In filtering an evidence map by outcome features, exposures which behave in a similar manner can be identified and investigated further for chemical similarity and/or shared modes of action. This information can be used to group substances, such that data-rich members of the group can be used to make predictions about data-poor members, without pursuing further primary research (Vink et al., 2010). Conversely, filtering an evidence map by chemical group or structural similarity may allow identification of shared outcomes, of similar relevance to read-across applications.

### 5.4. Evidence surveillance

Once regulation is in place, it is vital that it is kept up to date. Such is the role of the ongoing, evidence surveillance phase of regulatory decision-making. Within REACH, registrants are required to update their registration dossiers “whenever new information is available” (European Chemicals Agency, 2017b), such that dossiers are living products. However, a report commissioned by the European Chemicals Agency (ECHA) found that 64% of REACH registration dossiers submitted to ECHA since 2008 have never been updated (Amec Foster Wheeler Environment and Infrastructure UK Limited, 2017). The report details several obstacles experienced by registrants faced with updating dossiers, including technical difficulties, issues of ownership or responsibility for updates among co- and lead registrants, the potentially labour-intensive nature of updating dossiers and a perception of REACH registration being the “end of a process”.

Openly accessible and easily updated SEMs may serve to address such obstacles. As the population of a SEM database does not require detailed analysis or complex interpretation of the raw data, SEMs could be amenable to automation. Technological advances in text-mining and artificial intelligence might assist the automatic screening, extraction and coding of new information as it is published, based on the data fields and coding ontologies used to populate the original SEM. Although some years away from implementation, application of SEM methodology in the interim will promote fast uptake of such technological advances.

## 6. Conclusion

Systematic evidence mapping presents a transparent and robust methodological framework with which to assess the evidence landscape at the level of individual chemical risk management and innovation, to regulatory decision-making in chemicals policy. The broad scope of SEMs lowers the barrier to evidence synthesis in chemical risk assessment through more efficient use of resources. Future developments in text mining and machine learning are likely to further reduce the resource intensity of the methodology, and of chemical risk assessment in general. These advances will enable the automatic production of highly resolved SEMs capable of synthesising evidence or feeding predictive models.

In the interim pursuit of a more evidence-based approach to chemicals policy, the resource strain associated with producing a SEM can be managed through adaptation of the methodology to present day limitations. Depending on the needs of the user and the constraints of their use case, SEM methodology is sufficiently flexible that it may be adapted (e.g. by searching fewer databases, extracting data based on only title/abstract etc.) without compromising the utility of the end product in the same way as the results of a synthesis might be adversely affected by modification of systematic review methodology. By working closely with stakeholders to define objectives, the scope of the SEM (i.e. bibliographic databases covered, types of studies included, etc.) can be adjusted as appropriate to objectives. For example, critical appraisal of studies may not be imperative to the aim of the SEM and may therefore be omitted or might be planned as part of a stepwise approach after the SEM identifies pockets of evidence of interest to stakeholders. Although designed to reduce the resource strain of SEM exercises, such flexible adaptation of the methodology does not compromise the fitness-for-purpose of SEMs as a means of identifying and comparing trends in the availability of evidence in a vast and heterogeneous information landscape.

Consequently, examples of research activities producing fit-for-purpose SEM outputs and/or developing aspects of SEM methodology specific to chemicals policy contexts are beginning to emerge (Beverly, 2019), with research institutes such as NTP-OHAT and The Endocrine Disruption Exchange (TEDX) conducting evidence mapping activities (NTP-OHAT, 2019; The Endocrine Disruption Exchange, 2019). A key consideration for these emerging efforts is the accessibility of SEMs’ queryable output for non-technical audiences. To this end, researchers have made use of a variety of readily available and user-friendly tools (e.g. Datawrapper GmbH, 2019; IBM, 2019; QlikTech International AB, 2019; Tableau Software, 2019 etc.) to facilitate visualization of, and promote interaction with, the data collated in evidence surveillance exercises (e.g. Pelch et al., 2019; Walker et al., 2018). These tools may similarly serve to lower the barrier to accessing (as well as producing) SEMs, provided the underlying database is made available for more specialist users. Although future technological advances will have significant implications for the production and use of SEMs, these efforts indicate how SEM methodology can be effectively applied in present day, highlighting how SEMs can be adapted for engaging with a variety of stakeholders. More immediate establishment of (adapted) SEM infrastructure in current regulatory workflows will therefore not only lower resource barriers to evidence-based decision-making, but will ensure that technological advances in automation, and in SEM methodology itself, can be readily exploited by regulatory decision-makers in chemicals risk management.

## Figures and Tables

**Table 1:** The key features of systematic reviews and their primary advantages.

Systematic review step	Primary advantages
Pre-published protocol	Reduces risk that expectation bias will influence reviewers’ choice of methods and approaches for analysis mid-review; if formally published, external peer review can reduce risk of limitations in planned methods from compromising final results.
Statement of objectives	Provides a structured framework for the aims of the review (including specific statement of the research question and PECO criteria) against which appropriate review methods can be defined.
Comprehensive search	Reduces risk of only partial retrieval of the overall body of evidence that is relevant to answering the research question.
Screening against eligibility criteria (study inclusion)	Reduces risk of only partial retrieval of the overall body of evidence that is relevant to answering the research question, in particular the risk of selection bias when reviewers are deciding which evidence to include in the review.
Data extraction using appropriate extraction tools	Reduces risk of inconsistent or partial retrieval of data from studies included in the review, reducing risk of selective use of data from studies deemed relevant to answering the research question.
Critical appraisal of included studies	Encourages consistent assessment of validity of included studies according to factors internal to study design, reducing risk of expectation bias or other factors causing studies to be inappropriately weighted, and helping ensure that bias in the findings of the included studies is not transmitted through to the findings of the review.
Synthesis of included studies	Pooling or integration of sufficiently comparable studies increases the power of an analysis, whether quantitative or qualitative, allowing overall trends in results to be more reliably identified.
Characterisation of confidence in the evidence	Encourages consistent assessment of the validity of the results of the synthesis according to features which manifest at the level of body of evidence as a whole rather than the individual study. Outlining the scientific judgement applied in rating confidence is key to the transparency of subsequent conclusions.
Drawing conclusions/key review output	Qualitative and/or quantitative summary effect estimates help direct policy decisions based on permissible exposure levels and related controls; assessment of limitations in the review methods helps ensure that any residual potential biases in the review are made clear to the reader and can additionally be accounted for in uncertainty assessment and consequent risk management action.

PECO = Population-Exposure-Comparator-Outcome.

**Table 2:** A comparison of systematic review and systematic evidence mapping methodology and their respective roles in risk management decision-making (adapted from James et al., 2016).

Step	Conduct of step in SRs related to assessing chemical health risks	Conduct of step in SEMs related to assessing chemical health risks	SR vs SEM for responding to risk management needs
Pre-published protocol	Define all methods in advance of conduct of review	Same	Provides transparency; reduces bias; opportunity for peer review and stakeholder engagement. Applies to both SRs and SEMs.
Statement of objectives	Question concerns the effect of an exposure on health; or the effect of intervening to reduce exposure in terms of health benefit. Usually targets a single or few exposures and outcomes.	Question concerns the state of the evidence base for a topic. Usually open-ended and encompassing a range of multiple related exposures and outcomes.	SR: Focused, closed questions of SRs best service specific RM decisions such as characterising specific health risks/TDIs. SEM: Open questions of SEMs best service scenarios in which evidence should be surveyed and scoped, such as problem identification and priority-setting.
Comprehensive search	Search terms highly resolved and specified for most key elements of the objective statement, returning a moderate volume of evidence.	Wide ranging search strings of lower specificity based on topic rather than defining all key elements of the objective in the search.	SR: Narrow searches efficiently identify evidence related to exposure-outcome pairs. Maximum feasible number of sources searched to ensure collation of all relevant evidence for synthesis. SEM: Broader, topic-based SEM search allows evidence supportive of multiple decision scenarios to be identified. Flexible number of sources searched, or sources searched in a step-wise manner as appropriate to broader research objectives.
Screening against eligibility criteria (study inclusion)	Inclusion criteria specified in detail for all key elements of the objective.	Inclusion criteria defined in terms of topic rather than key elements of the objective.	SR: As for search, specific inclusion criteria ensure SRs efficiently service a specific research question. SEM: Broad objectives ensure inclusion of evidence relating to multiple decision scenarios.
Data extraction using tested extraction sheets	Complete extraction of meta-data and study findings.	Extraction of meta-data; optional extraction of study findings and other study characteristics depending on SEM objectives.	SR: Data extraction determined by objectives. SEM: Data extraction more flexible and can respond to needs of risk management process to develop fit-for-purpose maps of varying degrees of comprehensiveness.
Coding of extracted data using controlled vocabularies	Coding facilitates grouping of included studies for synthesis/integration according to review objectives. Coding is closely related to review objectives and data extraction process, whereby narrow research question and PECO statement inherently define specific code applicable to raw extracted data.	Coding facilitates broad comparison of heterogeneous data across an evidence base. Broad map objectives necessitate extensive coding process, whereby specific code must be defined in a step distinct from the formulation of end-users’ specific research questions.	SR: Tight review objectives pre-specify applied code (e.g. considering ages 0–18 as ‘Child’ for reviews focusing on a population of ‘Children’). Narrower range, or greater specificity of controlled vocabulary terms applicable per item of extracted data. SEM: Code pre-specified where possible, but addition of new terms (which could not be accounted for *a priori*) considered flexible. Any one item of extracted data may be coded by multiple and variably resolved terms. Openly accessible ontologies may be used for coding to promote consistency and interoperability.
Critical appraisal of included studies	Assessment of internal validity (risk of bias) conducted for all included studies.	Study validity assessment is optional and to some extent restricted if outcome is not a defined aspect of the SEM; study characteristics relevant to risk of bias assessment can be extracted.	SR: Describe the internal validity of the evidence base, which is an essential step of characterising confidence in the evidence. SEM: Flexible, critical appraisal step can be omitted; study methods are mapped or methodological quality assessed to goals, can be part of stepwise approach where quality only assessed for studies addressing key outcomes etc.
Synthesis of included studies	Quantitative synthesis where possible to produce characterisation of hazard from exposure; qualitative synthesis where pooling studies is not possible.	Reports of systematic maps can provide narrative synthesis of characteristics of the evidence key to a given decision-making context.	SR: Synthesis supports a specific type of decision context. SEM: Primary output is a more context-agnostic database which can be used by risk managers to support multiple decisions in the RM workflow; or to aid in a stepwise approach.
Characterisation of confidence in the evidence	Assessment of confidence or certainty in the results of the synthesis, according to characteristics of the evidence base taken as a whole.	SEMs do not synthesise included studies. SEMs help identify regions of evidence with characteristics indicative of being worth further, detailed analysis in support of a prospective decision.	SR: Provide detailed conclusions on certainty of evidence in hazard characterisation or to support risk assessments. SEM: Support a range of decisions, particularly decisions to focus research and review, e.g. indicating clusters where evidence may be strong enough to warrant SR (e.g. have a reasonable likelihood of changing a TDI), fill in gaps to reduce uncertainty and for surveillance.
Drawing conclusions/key review outputs	SRs primarily provide a summary effect estimate and surrounding uncertainty based on strength of the evidence and review methods.	SEMs primarily provide a searchable database of the characteristics of the evidence base, making the knowledge base locked away in manuscripts accessible to decision-makers.	SR: provide a qualitative and/or quantitative summary effect estimate in answer to a narrow and specific decision-making question. SEM: identify evidence gluts for synthesis. When combined with an understanding of RM needs, transparent criteria for prioritization of gluts for synthesis and gaps for commissioning primary research can be presented.

SR = systematic review, SEM= systematic evidence map, RM =risk management, TDI= tolerable daily intake.
